# Stability Research Considering Non-Linear Change in the Machining of Titanium Thin-Walled Parts

**DOI:** 10.3390/ma12132083

**Published:** 2019-06-28

**Authors:** Haining Gao, Xianli Liu

**Affiliations:** 1 College of Mechanical and Energy Engineering, HuangHuai University, Zhumadian 463000, China; 2 School of Mechanical & Power Engineering, Harbin University of Science and Technology, Harbin 150080, China

**Keywords:** stability lobe diagram, milling, process-damping, dynamic characteristics, thin-walled weak rigidity parts

## Abstract

Aiming to solve the problem whereby the damping process effect is significant and difficult to measure during low-speed machining of titanium alloy thin-walled parts, the ploughing coefficient of the flank face is obtained based on the frequency-domain decomposition (FDD) of the measured vibration signal and the energy balance principle, and then the process-damping prediction model is obtained. Aiming to solve the problem of non-linear change of dynamic characteristics of a workpiece caused by the material removal effect in the machining of titanium alloy thin-walled parts, a prediction model of dynamic characteristics of a workpiece is established based on the structural dynamic modification method. Meanwhile, the effect of material removal on the process-damping coefficient is studied, and the internal relationship between the process-damping coefficient and the dynamic characteristics of the workpiece is revealed. The stability lobe diagram is obtained by the full discretization in the titanium alloy milling process. The correctness of the model and stability prediction is verified by experiments under different working conditions. It is found that the coupling characteristics of process-damping and workpiece dynamic characteristics control the stability of the milling process. The research results can provide theoretical support for accurate characterization and process optimization of titanium alloy thin-walled workpiece milling.

## 1. Introduction

Titanium alloy thin-walled parts have found an increasingly wide use in the aviation manufacture industry because they can meet the requirements of high performance and high stability. Due to the low stiffness and high material removal rate, chattering occurs very easily in the machining process, which reduces the surface precision of the workpiece and the service life of cutters and machine tools, limiting production efficiency. It has become an urgent problem to be solved in the aviation manufacturing industry.

Titanium alloys are mostly machined at low speed. In the process of low-speed machining, the periodic friction and extrusion between the tool flank and the machined surface will produce a ploughing effect, which improves the processing stability [1,2]. Sisson et al [3] and Peters et al [4] studied the process-damping phenomenon in a low-speed cutting process with an experimental method. It was found that the ultimate cutting depth obtained by considering process-damping has been effectively improved, especially in the low-speed range. Wu et al [5] constructed a ploughing force model which can accurately characterize the process-damping. It was assumed that ploughing force was a linear function of the total volume of the cutter–worker interference and ploughing coefficient. Subsequently, researchers have conducted in-depth research on the accurate acquisition of the total volume of cutter–worker interference and ploughing coefficient. Lee et al [6] solved the relative vibration of tool–workpiece based on an iterative algorithm, and then obtained the total volume of tool–workpiece interference. The total volume of cutter–workpiece interference was calculated based on a numerical algorithm [7]. Cao et al [8] proposed two integration methods to calculate the cutter–worker interference area. The identification of ploughing coefficient is as follows. Wu et al [5] calculated ploughing coefficient by the contact theory. Shawky et al [9] identified ploughing coefficient by using the relationship between measured normal cutting force and static interference volume function. Tun et al [10] proposed a method for identifying process-damping coefficients directly by experimental stability limits. Ahmadi et al [11] presented a method to obtain the average process-damping coefficient of a dynamic cutting system by steady cutting experiments. Wan et al [12] provided a new method to identify process-damping based on the model proposed by Ahmadi and a modal analysis method. In addition, Jin et al [13] modeled process-damping through finite-element simulation of the vibration tool. Feng et al [14] found that the cutting vibration is the key factor affecting process-damping. However, there is no consensus on the analysis of the process-damping effect. 

The inherent properties of the workpiece (modal mass, stiffness, and damping ratio) change with the material removal, especially for the processing of weak rigid parts. This material removal effect makes it more difficult to accurately model the dynamic characteristics of the process system. The finite-element method (FEM) and the structural modification method (SDM) are often used to study material quality removal effect. Adetoro et al [15], Song et al [16], and Campa et al [17] used a two-dimensional finite-element model to study the variation of workpiece modal parameters with the processing location and material removal. Combining with the stability solution method (frequency-domain method and semi-discrete method), the stability lobe diagrams under the corresponding working conditions were obtained. Ding et al [18] proposed a 3D FEM model to predict the modal parameters of each machining step. FEM can effectively raise the prediction accuracy when the cutting step is reduced, but it also reduces the processing efficiency. Besides, FEM cannot simulate the condition of small radial depth of cut. The structural modification method was proposed by Özgüven et al [19] in 1990. The main idea is to calculate the frequency-response function of the modified system by using the frequency-response function of the original system and modifying the dynamic structure matrix of the system. Salih et al [20] and Budak et al [21] obtained milling stability lobe diagrams with material removal based on structure-modification method and frequency-domain method. Song et al [22] developed a new method for dynamic modification of equal mass structure to predict the variation of inherent properties of workpiece with material removal. 

Accurate acquisition of material mass-removal effect and process-damping effect is an effective way to enhance the stability prediction accuracy in the machining of titanium alloy. With the removal of material, the vibration resistance of the workpiece decreases, and the cutting vibration amplitude increases with the same cutting parameters. The process-damping coefficient is affected by the amplitude of vibration [10]. The interaction mechanism between material removal effect and process-damping has not been revealed in the existing literature. Meanwhile, tool stiffness in the cross-feed direction is lower than that of the workpiece-clamping system. The traditional two-degree-of-freedom dynamic equation of workpiece or cutting system is not applicable under this condition. In this paper, a new general dynamic model of the dynamic characteristics of the workpiece and process-damping is established. The milling stability is obtained by discrete method, and the effects of workpiece dynamic characteristics and process-damping on the stability are investigated. The research results can provide theoretical support for accurate characterization and process optimization of titanium alloy thin-walled workpiece milling.

## 2. Defining a Dynamic Model for Process-Damping

**Figure 1:**
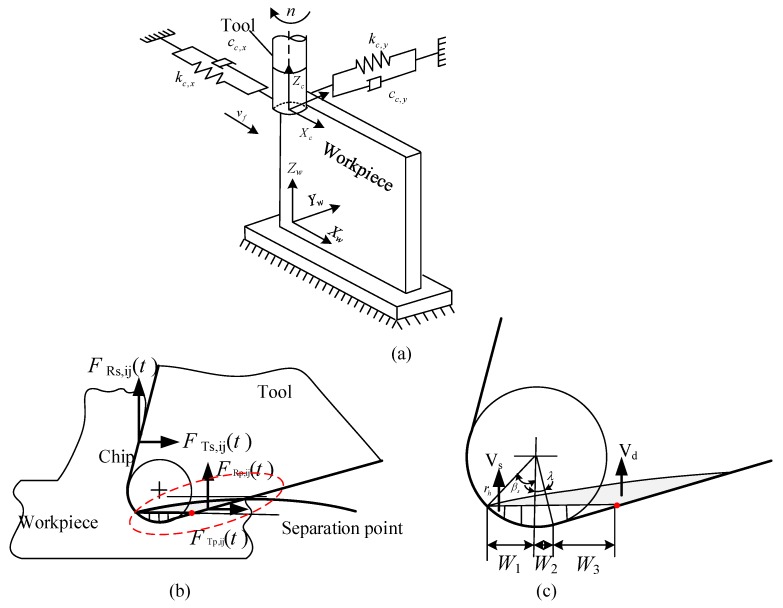
Dynamic model of milling system. (**a**) Three-dimensional model, (**b**) Schematic of chip generation, (**c**) Area of the indented volum.

The workpiece has weak rigidity in the normal (*Y*) direction, and the tool has weak rigidity in the feed (*X*) direction in in the machining of titanium alloy, as shown in Figure 1. Therefore, a two-degree-of-freedom dynamic model is founded in this work, which considered the vibration characteristics of the workpiece and the tool. The shear force is produced on the rake face due to shear effect, and the ploughing force is produced on the flank face due to friction and extrusion effect in the process of cutting material. The shear force and ploughing force can be broken into two sections—the static milling force and the ploughing milling force. Ignoring the modal coupling effect of the process system, the dynamic equation of cutting process is established, as shown in Equation (1).

(1) $$\left\{ \begin{array}{l} \left[m_{xx}]\{\ddot{x}(t)\}+[c_{xx}]\{\dot{x}(t)\}+[k_{xx}]\{x(t)\}=\{F_{x}(t)\right\}\\ \left[m_{yy}]\{\ddot{y}(t)\}+[c_{yy}]\{\dot{y}(t)\}+[k_{yy}]\{y(t)\}=\{F_{y}(t)\right\} \end{array}\right.$$

$\left[m_{xx}\right]$, $\left[c_{xx}\right]$, $\left[k_{xx}\right]$ are the mass, structural damping, and stiffness matrices of the cutting system, respectively. $\left[m_{yy}\right]$, $\left[c_{yy}\right]$, $\left[k_{yy}\right]$ are the mass, structural damping, and stiffness matrices of the workpiece, respectively. $\left\{F_{i}(t)\right\}$ is the cutting force in the *X* and *Y* directions. {i(t)} is the response caused by the cutting force. i=x or y.

The calculation formula of the cutting force in Y direction is shown in Equation (2).

(2) $$\left\{F_{y}(t)\}=F_{ys,st}(t)+F_{ys,dy}(t)+F_{yp,st}(t)+F_{yp,dy}(t\right)$$

$F_{ys,st}(t)$ and $F_{yp,st}(t)$ are the static cutting forces produced by shearing and friction on rank and flank face. $F_{ys,dy}(t)$ and $F_{yp,dy}(t)$ are the dynamic cutting force produced by shearing and friction on rank and flank face.

Static cutting force has no influence on the regeneration effect of machining process, so it is often ignored in the process of dynamic modeling.

The dynamic equation of processing is obtained as shown in Equation (3).

(3) $$\left[m_{yy}]\{\ddot{y}(t)\}+[c_{yy}]\{\dot{y}(t)\}+[k_{yy}]\{y(t)\}=F_{ys,dy}(t)+F_{yp,dy}(t\right)$$

(4) $$\left\{ \begin{array}{l} F_{ys,dy}(t)=g(\phi_{i,j})\sum_{j=0}^{N-1}\left(\sin(\phi_{i,j})F_{tsj}(t)-\cos(\phi_{i,j})F_{rsj}(t)\right)\\ F_{tsj}(t)=K_{Ts}a_{p}h(\phi_{i,j})\\ F_{rsj}(t)=K_{Rs}a_{p}h(\phi_{i,j})\\ h(\phi_{i,j})=\Delta x\sin(\phi_{i,j})+\Delta y\cos(\phi_{i,j}) \end{array}\right.$$

(5) $$\left\{ \begin{array}{l} F_{yp,dy}(t)=g(\phi_{i,j})\sum_{j=0}^{N-1}\left(\sin(\phi_{i,j})F_{tpj}(t)-\cos(\phi_{i,j})F_{rpj}(t)\right)\\ F_{tpj}(t)=K_{Tf}V_{d,j}\\ F_{rpj}(t)=K_{Rf}V_{d,j}\\ V_{d,j}=\frac{l_{w}^{2}}{2v_{c}}(\dot{x}\sin(\phi_{i,j})+\dot{y}\cos(\phi_{i,j})) \end{array}\right.$$

$g(\phi_{i,j}(t))$ is a window function for judging the processing state. Its expression is shown in Equation (6). $K_{Ts}$ and $K_{Rs}$ are the shear force coefficients on the rake face. $K_{Tf}$ and $K_{Rf}$ are the ploughing force coefficients on the flank face.

(6) $$g(\phi_{i,j}(t))=\{\begin{array}{c} 1,\;if\phi_{st}\leq \theta_{i,j}\leq \phi_{ex}\\ 0,\;otherwise \end{array}$$

$l_{w}$ is the boundary length between static indented and dynamic indented volumes.

(7) $$l_{w}=W_{1}+W_{2}+W_{3}$$

The angle of cutting rotation for the cutter tooth is $\phi_{i,j}(t)$. The expression of the tool rotation angle considering the tool helix angle, is shown in Equation (8).

(8) $$\phi_{i,j}=\frac{2\pi \Omega t}{60}+\frac{2\pi (j-1)}{N}-\frac{dz\tan\beta }{R}$$

where Ω is the spindle speed, N is the number of cutter teeth, β is the helix angle of the cutting tool, dz is the length of the axial unit.

Similarly, the dynamic shear force $F_{xs,dy}(t)$ and the dynamic ploughing force $F_{xp,dy}(t)$ in the X direction are obtained. We can get the dynamic equations with equivalent process-damping as follows.

(9) $$\begin{array}{l} \left[\begin{array}{cc} m_{xx} & 0\\ 0 & m_{yy} \end{array}\right]\left\{\begin{array}{c} \ddot{x}\\ \ddot{y} \end{array}\right\}+\left[\begin{array}{cc} k_{xx} & 0\\ 0 & k_{yy} \end{array}\right]\left\{\begin{array}{c} x\\ y \end{array}\right\}+\left(\left[\begin{array}{cc} c_{xx} & 0\\ 0 & c_{yy} \end{array}\right]+\left[\begin{array}{cc} c_{px} & 0\\ 0 & c_{py} \end{array}\right]\right)\left\{\begin{array}{c} \dot{x}\\ \dot{y} \end{array}\right\}\\ \;=\frac{1}{2}a_{p}\left[\begin{array}{cc} a_{xx} & a_{xy}\\ a_{yx} & a_{yy} \end{array}\right]\left\{\begin{array}{c} \Delta x\\ \Delta y \end{array}\right\} \end{array}$$

(10) $$\left\{\begin{array}{c} c_{px}\\ c_{py} \end{array}\right\}=\frac{l_{w}^{2}}{2v_{c}}\left[\begin{array}{cc} -\cos(\phi_{i,j}) & -\sin(\phi_{i,j})\\ \sin(\phi_{i,j}) & -\cos(\phi_{i,j}) \end{array}\right]\left[\begin{array}{cc} K_{Tf}\sin(\phi_{i,j}) & K_{Tf}\cos(\phi_{i,j})\\ K_{Rf}\sin(\phi_{i,j}) & K_{Rf}\cos(\phi_{i,j}) \end{array}\right]$$

(11) $$\{\begin{array}{c} a_{xx}=\sum_{j=0}^{N-1}-g(\phi_{i,j})[\sin(2\phi_{i,j})K_{Ts}+(1-\cos(2\phi_{i,j}))K_{Rs}]\\ a_{xy}=\sum_{j=0}^{N-1}-g(\phi_{i,j})[(\cos(2\phi_{i,j})+1)K_{Ts}+\sin(2\phi_{i,j})K_{Rs}]\\ a_{yx}=\;\sum_{j=0}^{N-1}g(\phi_{i,j})[(1-\cos(2\phi_{i,j}))K_{Ts}+\sin(2\phi_{i,j})K_{Rs}]\\ a_{yy}=\sum_{j=0}^{N-1}g(\phi_{i,j})[\sin(2\phi_{i,j})K_{Ts}-(\cos(2\phi_{i,j})+1)K_{Rs}] \end{array}$$

where $c_{px}$ and $c_{py}$ are the process-damping coefficients in machining process.

## 3. Prediction of Part Dynamics

The change of dynamic characteristics caused by material removal effect can be regarded as structural modification. Combining the original frequency-response function of the workpiece with the change value of the frequency-response function caused by material removal, the inherent properties of the workpiece after material removal can be obtained. The response of process system composed of workpiece and fixture under the cutting force in frequency domain can be expressed as:

(12) $$\left\{y(\omega )\}={\left[[k_{yy}]-\omega^{2}[m_{yy}]+\tau [c_{yy}]\right]}^{-1}\{F_{y}(\omega )\right\}$$

where τ is the unit imaginary number.

The receptance matrix, [α] is defined by:

(13) $$\alpha ={\left[[k_{yy}]-\omega^{2}[m_{yy}]+\tau [c_{yy}]\right]}^{-1}$$

The change of inherent properties of workpiece caused by material removal effect can be expressed as [ΔM], [ΔC] and [ΔK]. The dynamic structure-modification matrix is shown in Equation (14).

(14) $$\left[D]=[\Delta k_{yy}]-\omega^{2}[\Delta m_{yy}]+\tau [\Delta c_{yy}\right]$$

The receptance matrix is obtained by simultaneous Equation (13) and Equation (14).

(15) $$[\gamma ]={\left[[[k_{yy}]+[\Delta k_{yy}]]-\omega^{2}[[m_{yy}]+[\Delta m_{yy}]]+\tau [[c_{yy}]+[\Delta c_{yy}]]\right]}^{-1}$$

The modified receptance matrix of workpiece structure can be expressed as follows.

(16) $$\left[\gamma_{ab}\;\vdots \;\gamma_{ab}\right]=\left[\alpha_{ab}\;\vdots \;0\right]\left[[I]-[D]\left[\begin{array}{cc} \gamma_{bb} & \gamma_{bc}\\ \gamma_{cb} & \gamma_{cc} \end{array}\right]\right]$$

where $\alpha_{ij}$ and $\gamma_{ij}$ are submatrices of [α] and [γ] respectively.

## 4. Solving the Process-Damping Coefficient

**Figure 2:**
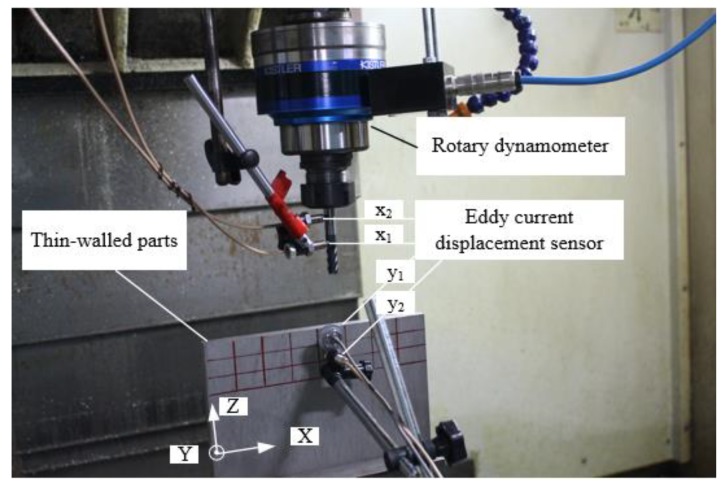
Experimental machining site.

Side milling is a discontinuous cutting style. Because of the inconvenience of the experimental acquisition equipment and the periodic change of the cutting signal, the process-damping coefficient cannot be obtained directly in milling experiments. In this paper, four eddy current displacement sensors are used to obtain the vibration signal of the workpiece and the cutting system during the machining process, as shown in Figure 2. Then the frequency-domain decomposition method is used to analyze the vibration signal of the process system, and the total damping coefficient of the process system is obtained. Finally, the ploughing coefficients of the process system are obtained.

The workpiece deformation *y(t)* in the *Y* direction can be expressed by the mode shape *U* and the mode displacement *q(t)*.

(17) $$\{\begin{array}{l} y(t)=Uq(t)\;\\ \begin{array}{l} y(t)={\left\{y_{1}(t)\;y_{2}(t)\right\}}^{T}\\ q(t)={\left\{q_{1}(t)\;q_{2}(t)\right\}}^{T}\\ U=\left[\begin{array}{cc} {\left\{\begin{array}{c} u_{1}\\ u_{2} \end{array}\right\}}_{1} & {\left\{\begin{array}{c} u_{1}\\ u_{2} \end{array}\right\}}_{1} \end{array}\right]=\left[\begin{array}{cc} {\left\{U\right\}}_{1} & {\left\{U\right\}}_{2} \end{array}\right] \end{array} \end{array}$$

Converting the power spectrum of the time domain deformation of experimental points y1, y2 into the frequency domain, the formula is as follows.

(18) $$S_{yy,[2\times 2]}=Y(j\omega )\cdot Y^{*T}(j\omega )$$

Y(jω) is the Fourier spectrum of workpiece deformation, ∗ and *T* represents the conjugate of a complex number and transpose of a matrix.

Equation (17) is introduced into Equation (18), and then Equation (18) is converted into Equation (19).

(19) $$S_{yy,[2\times 2]}=UQ(j\omega )Q^{*T}(j\omega )U^{*T}$$

Q(jω) is the Fourier spectrum of q(t).

**Figure 3:**
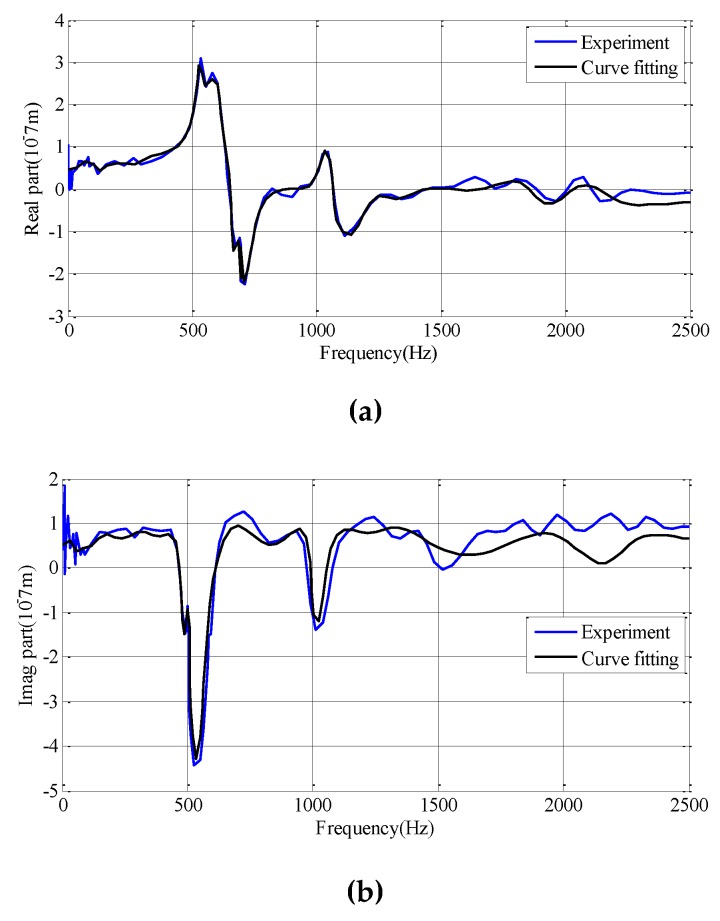
Fitting the measured frequency-response function.

$Q(j\omega )Q^{*T}(j\omega )$ is the 2*2 matrix. Due to the orthogonality of the modal, the asymmetry element is 0. In addition, because the modal of the system can be well separated (as shown in Figure 3), the Equation (19) can be simplified as follows.

(20) $$S_{yy,[2\times 2]}\approx U_{1}Q_{1}Q_{1}^{*T}U_{1}^{*T}$$

The magnitude of direct frequency-response functions (FRF) at any point can be obtained by the ratio of the power spectrum of the dynamic displacement of the point to the power spectrum of the exciting force. Therefore, the frequency-response function of position 1 is calculated as shown in Equation (21).

(21) $$\frac{S_{11}(j\omega )}{S_{ff}}={\left|H_{11}\right|}^{2}$$

where $S_{ff}$ is a fixed value [23].

The power spectrum of the deflection at point y1 can be transformed into the following form.

(22) $$S_{11}(j\omega )=u_{1}Q_{1}Q_{1}^{*T}u_{1}^{*T}$$

When the frequency range is the same, the frequency-response function can be expressed by modal parameters.

(23) $$\left\{ \begin{array}{l} H_{11}=\frac{1/m_{yy}}{\omega_{n,y}^{2}-\omega^{2}+2\xi_{r}\omega \omega_{n,y}j}\\ {\left|H_{11}\right|}^{2}=\frac{(1/m_{yy}{)}^{2}}{{\left(\omega_{n,y}^{2}-\omega^{2}\right)}^{2}+{\left(2\xi_{r}\omega \omega_{n,y}\right)}^{2}} \end{array}\right.$$

Equations (22) and (23) are substituted into Equation (21), the position sensitive detector (PSD) of mode coordinate q1 is obtained according to the modal parameters of the main mode.

(24) $$Q_{1}Q_{1}^{*}=\frac{S_{ff}/u_{1}u_{1}^{*}m_{r,y}^{2}}{{\left(\omega_{n,y}-\omega^{2}\right)}^{2}+{\left(2\xi_{r}\omega \omega_{n,y}\right)}^{2}}$$

Inverse Fourier transform for Equation (24).

(25) $$\left\{ \begin{array}{l} F^{-1}(Q_{1}Q_{1}^{*})=C_{2}e^{-\xi_{r,y}\omega_{n,y}t}\cos(\omega_{d}t)\\ C_{2}=\frac{S_{ff}/u_{1}u_{1}^{*}m_{r,y}^{2}}{2\omega_{{}_{n,y}}^{3}\xi_{r,y}\sqrt{1-\xi_{r,y}^{2}}}\\ \omega_{d}=\omega_{n,y}\sqrt{1-\xi_{r,y}^{2}} \end{array}\right.$$

The extreme points occur at $t=i\pi /\omega_{d}$. The absolute value of $F^{-1}$ can be expressed as a linear function. Substituting $t=i\pi /\omega_{d}$ in Equation (25), we get the peaks and valleys of $F^{-1}$.

(26) $$\left|P_{x,i}\right|=2C_{2}^{2}e^{\frac{-2i\pi \xi_{r,y}}{\sqrt{1-\xi_{{}_{r,y}}^{2}}}}$$

Logarithmic operation is conducted to linearize Equation (26).

(27) $$\left\{ \begin{array}{l} 2\ln\left|P_{x,i}\right|=\delta_{0}+\delta_{1}i\\ \delta_{0}=2\ln C_{2}\\ \delta_{1}=-\frac{2\pi \xi_{r,y}}{\sqrt{1-\xi_{{}_{r,y}}^{2}}} \end{array}\right.$$

The total damping ratio can be calculated by Equation (2).

(28) $$\xi_{r,y}=\frac{-\delta_{1}}{\sqrt{\delta_{{}_{1}}^{2}+4\pi^{2}}}$$

It is impractical to obtain the frequency-response function in machining process, so the autocorrelation of q1 is resolved by the vibration signal measured in cutting process.

The power spectrum matrix of the workpiece vibration measured at points y1, y2 can be expressed as follows.

(29) $$\begin{array}{l} S_{yy,[2\times 2]}^{e}(j\omega_{i})=Y_{e}(j\omega_{i})\cdot Y_{e}{\left(j\omega_{i}\right)}^{*T}\\ \;=\left[{\left\{V\right\}}_{1}\;{\left\{V\right\}}_{2}\right]\left[\begin{array}{cc} \kappa_{1} & 0\\ 0 & \kappa_{2} \end{array}\right]{\left[{\left\{V\right\}}_{1}\;{\left\{V\right\}}_{2}\right]}^{T}\\ \;={\left\{V\right\}}_{1}\kappa_{1}{\left\{V\right\}}_{{}_{1}}^{T}+{\left\{V\right\}}_{2}\kappa_{2}{\left\{V\right\}}_{{}_{2}}^{T} \end{array}$$

where $\omega_{i}$ is the frequency line. ($\kappa_{1}$, $\kappa_{2}$), (${\left\{V\right\}}_{1}$, ${\left\{V\right\}}_{2}$) are the eigenvalues and normalized eigenvectors of $S_{yy,[2\times 2]}^{e}(j\omega_{i})$ matrix, respectively. If $\kappa_{1}>\kappa_{2}$ near the main mode, Equation (29) can be simplified as follows.

(30) $$S_{yy,[2\times 2]}^{e}(j\omega_{i})\approx {\left\{V\right\}}_{1}\kappa_{1}{\left\{V\right\}}_{{}_{1}}^{T}$$

The similarity between eigenvectors and modal shapes is determined on each frequency line using modal assurance criteria (MAC).

(31) $$MAC(\omega_{i})=\frac{\left|{\left\{U\right\}}_{1}^{T}\cdot {\left\{V\right\}}_{1}^{T}\right|}{\left({\left\{U\right\}}_{1}^{T}\cdot {\left\{V\right\}}_{1})\cdot ({\left\{U\right\}}_{1}^{T}\cdot {\left\{V\right\}}_{1}\right)}$$

When the value of $MAC(\omega_{i})$ is close to 1, $Q_{1}Q_{1}^{*}\approx \kappa_{1}$.

The difference between the total damping coefficient and the structural damping coefficient is used to calculate the process-damping coefficient.

(32) $$c_{p,y}=2m_{yy}\omega_{n,y}\xi_{r,y}-c_{s,y}$$

In the same way, the X-directional process-damping coefficient can be solved by Equation (33).

(33) $$c_{p,x}=2m_{xx}\omega_{n,x}\xi_{r,x}-c_{s,x}$$

The energy of the average process-damping effect is equal to the energy consumed by the dynamic plough force in the rotational period of the spindle, thus the ploughing force coefficients can be obtained.

(34) $$\left\{ \begin{array}{l} \int_{0}^{T}\left[\sum_{i,j}g(\phi_{i,j})\frac{l_{w}^{2}}{2v_{c}}\cos(\phi_{i,j})(-\cos(\phi_{i,j})K_{Tf}-\sin(\phi_{i,j})K_{Rf})dz_{i,j}\right]\dot{x}(t)dt=\int_{o}^{T}-c_{p,x}{\dot{x}}^{2}(t)dt\\ \int_{0}^{T}\left[\sum_{i,j}g(\phi_{i,j})\frac{l_{w}^{2}}{2v_{c}}\cos(\phi_{i,j})(\sin(\phi_{i,j})K_{Tf}-\cos(\phi_{i,j})K_{Rf})dz_{i,j}\right]\dot{y}(t)dt=\int_{o}^{T}-c_{p,y}{\dot{y}}^{2}(t)dt \end{array}\right.$$

## 5. Experimental

### 5.1. Experimental Setup

The experiment was carried out on a three-axis machining center manufactured by Dalian machine tool(Dalian machine tool group, Dalian, Liaoning, China). Its model is VDL-1000E. Solid carbide cutting tools with four teeth, diameter 10 and helix angle 30° are used for processing thin-walled titanium alloy parts. Its model is GM-4E-D10.0. The coating material is TiAlN. The extended length of the cutting tool is 110 mm. The geometric dimension of thin-walled titanium alloy parts is 200 × 200 × 5 mm. The workpiece is fixed on the vise. The vice is bolted to the workbench. The extended length of the workpiece is 100 mm. The milling mode is down milling and dry cutting. The rotary dynamometer produced by Kistler Company (Winterthur, Switzerland) was used to collect cutting force data. Its model is 5236B. Three-component acceleration sensors produced by PCB Sensor Company was used to collect the acceleration data of cutting process. Its model is 368F. The three-dimensional sensitivity is 2.462 mv/g, 2.534 mv/g and 2.487 mv/g, respectively. The initial modal parameters of the cutting system and workpiece are obtained by using the unidirectional acceleration sensor produced by PCB Sensor Company and the impact hammer. The vibration signals of the workpiece and the tool are measured using four ST-2-U-05-00-20-KH07 eddy current sensors. The experimental machining site is shown in Figure 2.

### 5.2. The Acquisition of Dynamic Characteristics

Modal parameters of cutting system are usually obtained by hammer impact experiment. Generally, the real part and imaginary part of the frequency-response function are fitted by curve fitting method to obtain the modal parameters of the process system, as shown in Figure 3. Machining process is mainly controlled by first-order modal parameters. The initial first-order modal parameters of the unmachined workpiece and the tool are shown in Table 1.

**Figure 4:**
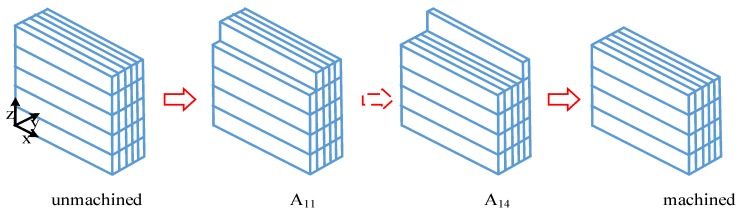
Schematic diagram of machining process.

The thickness of thin-walled titanium alloy parts is reduced from 5 mm to 0 mm by machining along the wall thickness. The process diagram is shown in Figure 4. The comparison results between the experimental and predicted values of the modal parameters of the workpiece at different machining positions is shown in Table 2.

Table 2 showed that with the removal of the material, the natural frequency of the workpiece increased, and the stiffness and damping ratio decreased gradually. The vibration resistance of workpiece is weakened. In addition, the changes of them displayed a non-linear feature. Therefore, if the damping ratio is assumed to be a fixed value or an equal proportion change, the prediction of dynamic force and stability will deviate. 

By comparing the predicted value and measured value of the modal parameters of the workpiece, it can be seen that the maximum error of natural frequency, stiffness and damping ratio is 10.07%, 8.85% and 7.90%, respectively. The error is within acceptable range to verify the accuracy of the workpiece dynamic model.

### 5.3. Ploughing Force Coefficient

**Figure 5:**
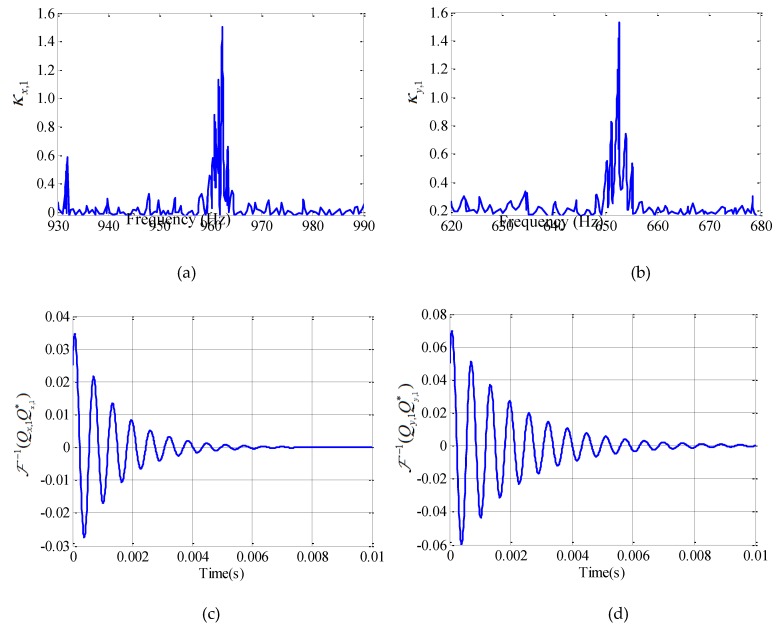

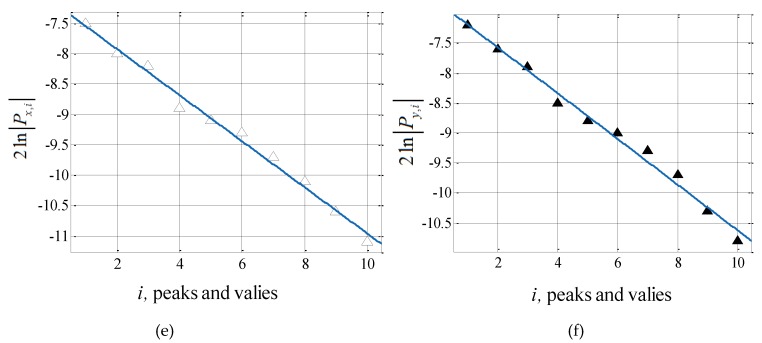
Identification of damping ratio in Z11. (**a**) Measured $\kappa_{x,1}$, (**b**) Measured $\kappa_{y,1}$, (**c**) $F^{-1}(Q_{x,1}Q_{{}_{x,1}}^{*})$, (**d**) $F^{-1}(Q_{y,1}Q_{{}_{y,1}}^{*})$, (**e**) Logarithmic decrement to identify damping, (**f**) Logarithmic decrement to identify damping in Y direction.

With *ap* = 6 mm, *ae* = 0.5 mm, *n* = 900 r/min, *f* = 0.1 mm/z, the steps to obtain the total damping ratio in the feed direction and normal direction of position A11 are shown in Figure 5.

The overall damping ratios are estimated by substituting the slope of the fitted lines in Equation (27), $\xi_{r,x}=0.0801$, $\xi_{r,y}=0.0798$.The process-damping coefficient is evaluated as $c_{p,x}=2m_{x}\omega_{n,x}(\xi_{r,x}-\xi_{s,x})=53.39(Ns/m)$, $c_{p,y}=2m_{y}\omega_{n,y}(\xi_{r,y}-\xi_{s,y})=31.09(Ns/m)$. The ploughing force coefficients are obtained to be $K_{Tf}=3.735\times 10^{13}N/m^{2}$, $K_{Rf}=1.208\times 10^{13}N/m^{2}$.

**Figure 6:**
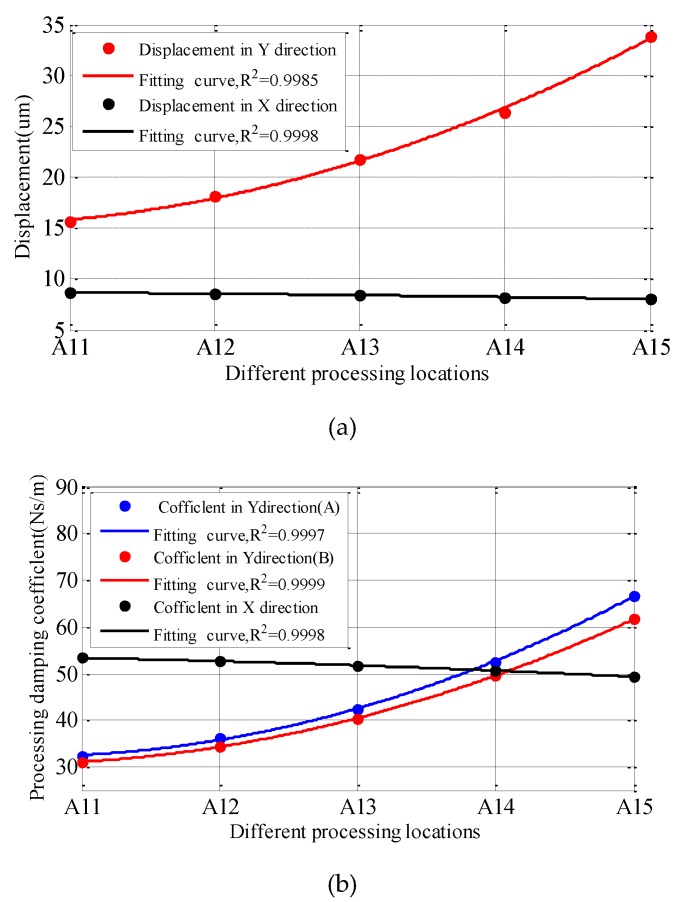
The effect of material removal effect on machining process. (**a**) Displacement, (**b**) Process-damping coefficients.

By analyzing the cutting vibration obtained by the cutting process shown in Figure 4, the variation law of the vibration amplitude and the process-damping coefficient with the material removal effect is obtained, as shown in Figure 6.

Figure 6a shows that under the same cutting parameters, the vibration amplitude of the machining system in the Y directions increases with the removal of workpiece materials. The change of vibration in the X direction is just the opposite to that in the Y direction. Meanwhile, it is found that the change rate of vibration amplitude in Y direction is greater than that in X direction.

It can be seen from Figure 6b that the process-damping coefficient in the Y directions increases with the removal of workpiece materials. The process-damping coefficient considering the dynamic characteristics of the workpiece (A) is smaller than that without considering the dynamic characteristics of the workpiece (B). The process-damping coefficient in the X directions decreases with the removal of workpiece materials.

## 6. Milling Stability

The frequency-domain method [24], discrete method and numerical method [25] are often used to solve the dynamic equation in machining process, and then then the stability of the cutting process is obtained. The frequency-domain method has the highest calculation efficiency, but the prediction accuracy is the lowest, especially it is not applicable to small radial depth of cut. The numerical method has the highest prediction accuracy, but its computational efficiency is the lowest because many equations need to be solved directly. The discrete method can be divided into the semi-discretization method [26], the full-discretization method [27] and the time FEM [28]. The semi-discretization method and the full-discretization method are most widely used. The matrix exponential function involved in the full-discretization method is only dependent on the spindle speed and independent of the axial depth of cut, so it is more efficient than the semi-discretization method. Therefore, we use the full discrete method to obtain the stability of the machining process.

The research results in Section 5.3 show that the process-damping coefficient has a non-linear relationship with the dynamic characteristics of the process system. Taking A15 machining position as an example, the influence of coupling characteristics between process-damping and dynamic characteristics of process system on milling stability is investigated. 

**Figure 7:**
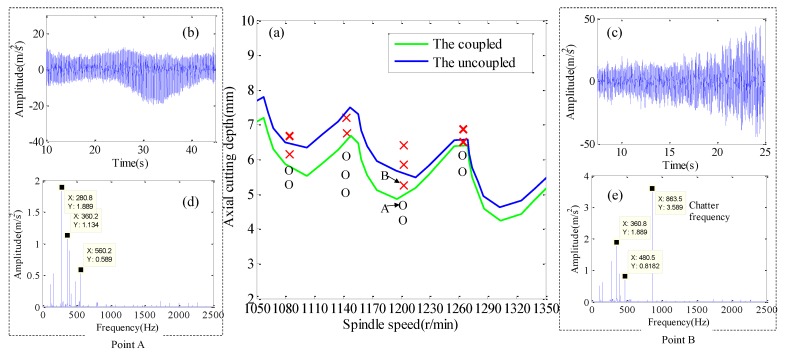
Validation of milling process stability. (**a**) Stability lobes diagram, (**b**) The acceleration signal at A point, (**c**) The acceleration signal at B point, (**d**) Frequency spectrum diagram at A point acceleration signal (**e**) Frequency spectrum diagram at B point acceleration signal.

The stability lobe diagrams with the coupled and the uncoupled process-damping, and the dynamic characteristics of the process system are shown in Figure 7a. A series of experiments (ae = 0.5 mm, f = 0.1 mm/tooth) were carried out to verify the accuracy of the stable lobe diagram. The remaining cutting parameters are detailed in Table 3. Two points A and B are selected for experimental verification to verify the correctness of the predicted results. Figure 7b,c are acceleration signals in time domain. Figure 7d,e are Fourier transform results of acceleration signals.

The maximum amplitude of point A acceleration signal is 18 m/s^2^, and its amplitude variation has better convergence, as shown in Figure 7b. Fourier transform is applied to the acceleration signal in time domain, and the result is shown in Figure 7d. It is found that the spectrum energy mainly concentrates on the cutter teeth passing frequency and its higher harmonics. The maximum amplitude of point B acceleration signal is 48 m/s^2^, and its amplitude change law is from small to large, which does not have convergence, as shown in Figure 7c. Fourier transform is applied to the acceleration signal in time domain, and the result is shown in Figure 7e. It is found that the spectrum energy is mainly concentrated near the first natural frequency of the workpiece.

**Figure 8:**
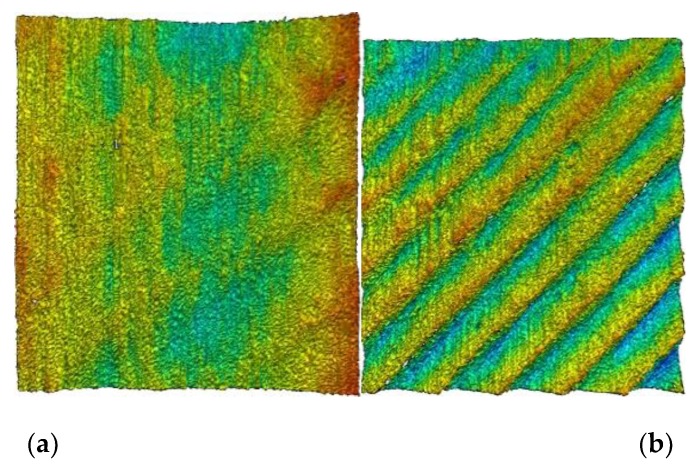

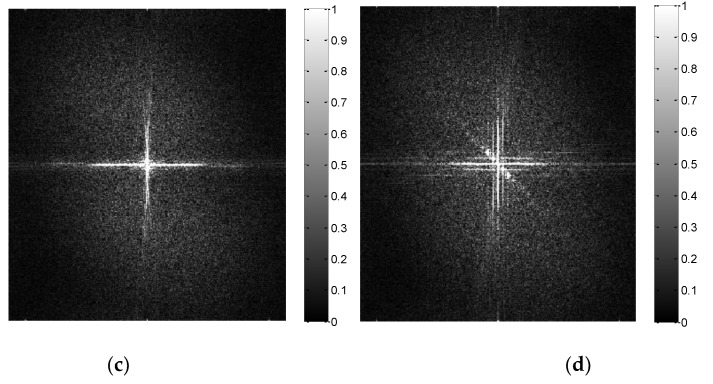
Microscopic morphology and frequency-domain analysis with A and B parameters. (**a**) Micromorphology at A point, (**b**) Micromorphology at B point, (**c**) 2DFFT at A point, (**d**) 2DFFT at B point.

Meanwhile, the white light interferometer is used to measure the micromorphology of machined surface with A and B parameters and the results are shown in Figure 8a,b. Two-dimensional Fourier transform (2DFFT) is applied to the image of micro-surface topography, and the results are shown in Figure 8c,d.

The machined surface obtained at point A has lower level of surface roughness (Sa = 0.85 um, Ra = 0.47 um) and surface waviness, as shown in Figure 8a. The only significant spectral property of the machined surface at point A is related to the marking belonging to feed-rate, as shown in Figure 8c. The machined surface obtained at point A has the higher level of surface roughness (Sa = 1.63 um, Ra = 1.03 um) and surface waviness, as shown in Figure 8b. The spectral characteristics of the surface topography generated by point B are inclined modes related to the chatter frequency, as shown in Figure 8d. In conclusion, A is the cutting stability point and B is the cutting chatter point. It is found that the stability obtained by considering the coupling characteristics of dynamic characteristics of workpiece and process-damping has higher prediction accuracy.

Milling stability of the coupled process-damping and dynamic characteristics of process system is lower than that of the uncoupled, as shown in Figure 7a. The reason is that the process-damping coefficient obtained from the coupling process-damping and the dynamic characteristics of the process system is small. Meanwhile, the difference between them decreases with the increase of spindle speed. The reason is that the damping coefficient descends with the increase of spindle speed.

**Figure 9:**
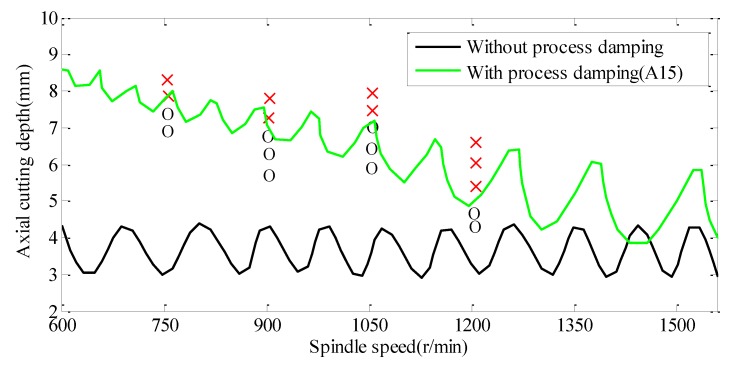
SLD, X-experimental stable, O-experimental unstable.

The stability lobe diagrams with and without process-damping are shown in Figure 9. A series of experiments (ae = 0.5 mm, f = 0.1 mm/tooth) were carried out to verify the accuracy of the stable lobe diagram. The remaining cutting parameters are detailed in Table 4. It is found that the milling stability considering process-damping is higher than that without process-damping in titanium alloy milling process, especially low spindle speed. The milling stability decreases with the increase of spindle speed. The reason is that the process-damping coefficient is inversely proportional to the spindle speed.

**Figure 10:**
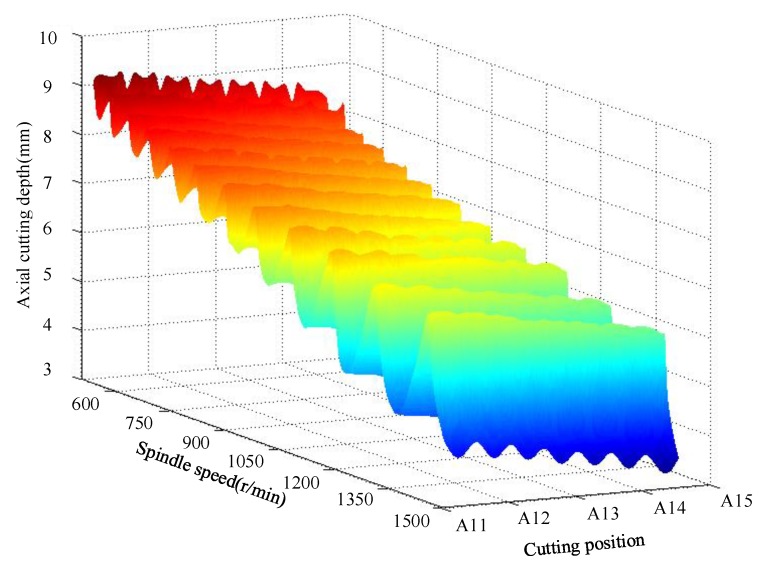
Three-dimensional stable lobe diagram.

The 3D stability lobe diagram in milling titanium alloy thin-walled parts is shown in Figure 10. It is found that the ultimate axial cutting depth decreases with material removal. The reason is that with the removal of the material, the natural frequency of the workpiece increased, and the stiffness and damping ratio decreased gradually. The vibration resistance of workpiece is weakened.

## 7. Conclusions

In this paper, two prominent problems in the processing of titanium alloy thin-walled parts—the non-linear changes of dynamic characteristics of the parts caused by material removal effect and the damping process effect is difficult to measure—are investigated in depth. Based on the frequency-domain decomposition of the measured vibration signal and the principle of energy balance, a process-damping prediction model is obtained. Based on the structural dynamic modification method, a prediction model of workpiece dynamic characteristics is established. The effect of material removal on process-damping coefficient is studied. The full discrete method is used to solve the stability of the milling process. The correctness of the model and stability prediction is verified by experiments with different working conditions. The research results can provide theoretical support for accurate characterization and process optimization of titanium alloy thin-walled workpiece milling. The specific conclusions are as follows:

(1) Under the same cutting parameters, the vibration amplitude of the workpiece increases with the material removal, which leads to the corresponding increase of the process-damping coefficient. The variation of vibration amplitude and damping coefficient of cutting system is just the opposite. 

(2) The process-damping coefficient obtained by considering the dynamic characteristics of the workpiece is less than that without considering the dynamic characteristics of the workpiece, and the gap between them increases with the removal of materials.

(3) Milling stability of the coupled process-damping and dynamic characteristics of process system is lower than that of the uncoupled. The reason is that the process-damping coefficient is relatively small. Meanwhile, the difference between them decreases with the increase of spindle speed. The reason is that the damping coefficient decreases with the increase of spindle speed.

(4) The milling stability is gradually reduced with the material is removed and the spindle speed is increased. Meanwhile, it is found that the effect of material removal on milling stability in the low-speed region is less than that in high speed region. The reason is that with the removal of materials, the vibration amplitude of the workpiece increases so that the damping coefficient of the process increases in the low-speed region, which further weakens the effect of material removal.

## Figures and Tables

**Table 1:** The initial first-order modal parameters.

Position	Natural Frequency (Hz)	Rigidity (N/m)	$\mathrm{Damping}\;\mathrm{Ratio}(\xi_{s})$
Tools (X direction)	963	4.85 × 10^7^	0.0591
Workpiece (Y direction)	652	8.54 × 10^6^	0.0310

**Table 2:** The modal parameters of the workpiece at different machining positions.

	Measured			Predicted		
Position	Natural Frequency (Hz)	Rigidity (N/m)	$\mathrm{Damping}\;\mathrm{Ratio}(\xi_{s,y})$	Natural Frequency (Hz)	Rigidity (N/m)	$\mathrm{Damping}\;\mathrm{Ratio}(\xi_{s,y})$
A11	675	8.47 × 10^6^	0.0308	612	7.82 × 10^6^	0.0287
A12	690	8.39 × 10^6^	0.0304	633	7.73 × 10^6^	0.0283
A13	718	8.30 × 10^6^	0.0299	647	7.68 × 10^6^	0.0279
A14	782	8.21 × 10^6^	0.0291	725	7.63 × 10^6^	0.0268
A15	864	8.11 × 10^6^	0.0275	789	7.59 × 10^6^	0.0259

**Table 3:** Machining state with different conditions.

	Cutting Parameters		Machining State		Cutting Parameters		Machining State
No.	N (r/min)	Ap (mm)		No.	N (r/min)	Ap (mm)	
1	1080	5.3	Stable	10	1200	4.3	Stable
2	1080	5.6	Stable	11	1200	4.7	Stable
3	1080	6.2	Chatter	12	1200	5.4	Chatter
4	1080	6.7	Chatter	13	1200	5.8	Chatter
5	1140	5.0	Stable	14	1200	6.5	Chatter
6	1140	5.5	Stable	15	1260	5.7	Stable
7	1140	6.0	Stable	16	1260	6.1	Stable
8	1140	6.6	Chatter	17	1260	6.3	Chatter
9	1140	7.2	Chatter	18	1260	6.8	Chatter

**Table 4:** Machining state with different conditions.

	Cutting Parameters		Machining State		Cutting Parameters		Machining State
No.	N (r/min)	Ap (mm)		No.	N (r/min)	Ap (mm)	
1	750	6.8	Stable	11	1050	6.4	Stable
2	750	7.2	Stable	12	1050	7.1	Stable
3	750	7.9	Chatter	13	1050	7.5	Chatter
4	750	8.2	Chatter	14	1050	8.0	Chatter
5	900	5.9	Stable	15	1200	4.3	Stable
6	900	6.3	Stable	16	1200	4.7	Stable
7	900	6.9	Stable	17	1200	5.4	Chatter
8	900	7.1	Chatter	18	1200	5.8	Chatter
9	900	7.5	Chatter	19	1200	6.5	Chatter
10	1050	6.0	Stable				

## References

[1] Yue C., Gao H., Liu X., Liang S.Y. (2019). A review of chatter vibration research in milling. Chin. J. Aeronaut.

[2] Liu X., Gao H., Yue C., Li R., Jiang N. (2018). Investigation of the milling stability based on modified variable cutting force coefficients. Int. J. Adv. Manuf. Technol..

[3] Sisson T.R., Kegg R.L. (1969). An explanation of low-speed chatter effects. J. Eng. Ind..

[4] Peters J., Panherck V., Brussel H.V. (1971). The measurement of the dynamic cutting coefficient. CIRP Ann.-Manuf. Technol..

[5] Wu W.D. (1989). A new approach of formulating the transfer function of dynamic cutting processes. J. Eng. Ind. (Trans. ASME).

[6] Lee B.Y., Tarng Y.S., Ma S.C. (1995). Modeling of the process damping force in chatter vibration. Int. J. Mach. Tool Manuf..

[7] Endres W.J., DeVor R.E., Kapoor S.G. (1995). A dual-mechanism approach to the prediction of machining forces, part 1: Model development. J. Eng. Ind..

[8] Cao C., Zhang X.M., Ding H. (2017). An improved algorithm for cutting stability estimation considering process damping. Int. J. Adv. Manuf. Technol..

[9] Shawky A.M., Elbestawi M.A. (1997). An enhanced dynamic model in turning including the effect of ploughing forces. J. Manuf. Sci. E-T ASME.

[10] TunL T., Budak E. (2013). Identification and modeling of process damping in milling. J. Manuf. Sci. E-T ASME.

[11] Ahmadi K., Ismail F. (2012). Stability lobes in milling including process damping and utilizing Multi-Frequency and Semi-Discretization Method. Int. J. Mach. Tool Manuf..

[12] Wan M., Feng J., Ma Y.C., Zhang W. (2017). Identification of milling process damping using operational modal analysis. Int. J. Mach. Tool Manuf..

[13] Jin X., Altintas Y. (2013). Chatter stability model of micro-milling with process damping. J. Manuf. Sci. E-T ASME.

[14] Feng J., Wan M., Gao T.Q., Zhang W. (2018). Mechanism of process damping in milling of thin-walled workpiece. Int. J. Mach. Tool Manuf..

[15] Adetoro O.B., Sim W.M., Wen P.H. (2010). An improved prediction of stability lobes using nonlinear thin wall dynamics. J. Mater. Process Technol..

[16] Song Q., Ai X., Tang W. (2011). Prediction of simultaneous dynamic stability limit of time-variable parameters system in thin-walled workpiece high-speed milling processes. Int. J. Adv. Manuf. Technol..

[17] Campa F.J., De Lacalle L.N.L., Celaya A. (2011). Chatter avoidance in the milling of thin floors with bull-nose end mills: model and stability diagrams. Int. J. Mach. Tool Manuf..

[18] Ding Y., Zhu L. (2018). Investigation on chatter stability of thin-walled parts considering its flexibility based on finite element analysis. Int. J. Adv. Manuf. Technol..

[19] Zgüven H.N. (1990). Structural modifications using frequency response functions. Mech. Syst. Signal Process.

[20] Alan S., Budak E., Zgüven H.N. (2010). Analytical prediction of part dynamics for machining stability analysis. Int. J. Autom. Technol..

[21] Budak E., TunL T., Alan S., Zgüven H.N. (2012). Prediction of workpiece dynamics and its effects on chatter stability in milling. CIRP Ann.-Manuf. Technol..

[22] Song Q., Liu Z., Wan Y., Ju G., Shi J. (2015). Application of Sherman-Morrison-Woodbury formulas in instantaneous dynamic of peripheral milling for thin-walled component. Int. J. Mech. Sci..

[23] Tunc L.T., Budak E. (2012). Effect of cutting conditions and tool geometry on process damping in machining. Int. J. Mach. Tool Manuf..

[24] Altintas Y., Budak E. (1995). Analytical prediction of stability lobes in milling. CIRP Ann..

[25] Smith S., Tlusty J. (1993). Efficient simulation programs for chatter in milling. CIRP Ann..

[26] Insperger T., Stépán G. (2004). Updated semi-discretization method for periodic delay-differential equations with discrete delay. Int. J. Numer. Method Eng..

[27] Bayly P.V., Halley J.E., Mann B.P., Davies M. (2003). Stability of interrupted cutting by temporal finite element analysis. J. Manuf. Sci. E-T ASM.

[28] Ding Y., Zhu L.M., Zhang X.J., Ding H. (2010). A full-discretization method for prediction of milling stability. Int. J. Mach. Tool Manuf..

